# Potential distribution of malaria vectors in Central Vietnam: A MaxEnt modeling approach

**DOI:** 10.14202/vetworld.2024.1514-1522

**Published:** 2024-07-13

**Authors:** Le Thanh Tam, Kavin Thinkhamrop, Sutas Suttiprapa, Apiporn T. Suwannatrai

**Affiliations:** 1 Department of Tropical Medicine, Faculty of Medicine, Khon Kaen University, Khon Kaen, Thailand; 2 Department of Epidemiology, Institute of Malariology, Parasitology, and Entomology Quy Nhon, Ministry of Health, Vietnam; 3 Health and Epidemiology Geoinformatics Research, Faculty of Public Health, Khon Kaen University, Khon Kaen, Thailand; 4 Department of Parasitology, Faculty of Medicine, Khon Kaen University, Khon Kaen, Thailand

**Keywords:** *Anopheles dirus*, *Anopheles minimus*, Central Vietnam, MaxEnt

## Abstract

**Background and Aim:**

In Central Vietnam, *Anopheles dirus* and *Anopheles minimus* are the primary malaria vector species. These *Anopheles* spp.’ distribution and prevalence are determined by environmental, climatic, and socioeconomic conditions. This study aimed to predict the potential distribution of these two *Anopheles* spp. in this region.

**Materials and Methods:**

This study was conducted in 15 Central Vietnamese provinces. From 2014 to 2018, we utilized *An. diru*s and *An. minimu*s presence records. Proxy data from the Google Earth Engine platform for the study area, encompassing environmental, climatic, and socioeconomic factors. MaxEnt software predicted the potential environmental, climatic, and socioeconomic suitability of these two *Anopheles* spp. in Central Vietnam.

**Results:**

The test area under the curve values for *An. dirus* and *An. minimus* MaxEnt models averaged 0.801 and 0.806, respectively, showing excellent performance. Minimum air temperature had the greatest impact on the distribution of both species. A negative correlation between precipitation and normalized difference water index influences the occurrence of *An. dirus*. In the temperature range of 13–19.5°C, *An. minimus* is most likely to be present, with nighttime light detrimentally influencing its distribution. The Central Highlands region is inhabited by both species, with some presence in North-Central and South-Central Coastal areas.

**Conclusion:**

The importance of temperature in determining the presence of both species is emphasized by our findings, with subtle differences in the temperature-related factors shaping their distributions. The results highlight the need for focused malaria vector control and surveillance initiatives in the study area.

## Introduction

Approximately 247 million global cases and 619,000 deaths were reported for malaria in 2021 [1]. In Southeast Asia, Vietnam bears a substantial malaria burden [2, 3]. In Central Vietnam’s varied landscapes, consisting of highlands, plains, and coasts, malaria control and prevention face distinct challenges [4, 5]. *Anopheles dirus* and *Anopheles minimus*, the major malaria vectors in Central Vietnam, transmit *Plasmodium* parasites [6, 7]. These *Anopheles* spp.’ distribution and prevalence are influenced by factors like temperature, precipitation (PREC), land cover, and socioeconomic conditions [8].

Effective vector control strategies and targeted interventions for reducing malaria transmission hinge on comprehending the spatial distribution of these vectors. MaxEnt modeling approach holds significant power in this context [9, 10]. MaxEnt, a machine learning technique, estimates species distribution probabilities using environmental variables [11, 12]. This method is commonly used in ecological and epidemiological studies to predict species distributions precisely [13–19].

This study aimed to determine *An. diru*s and *An. minimus*’ potential geographical distribution in Central Vietnam, considering current environmental, climatic, and socioeconomic conditions. The study’s findings will benefit scientific community’s knowledge of malaria vector distribution and inform policymakers and public health officials. These insights can effectively guide malaria interventions and resource allocation toward national malaria elimination in Central Vietnam.

## Materials and Methods

### Ethical approval

The Khon Kaen University Ethics Committee for Human Research approved and granted permission to use data for the study on September 20, 2023 (HE661434).

### Study period and location

**Figure-1:**
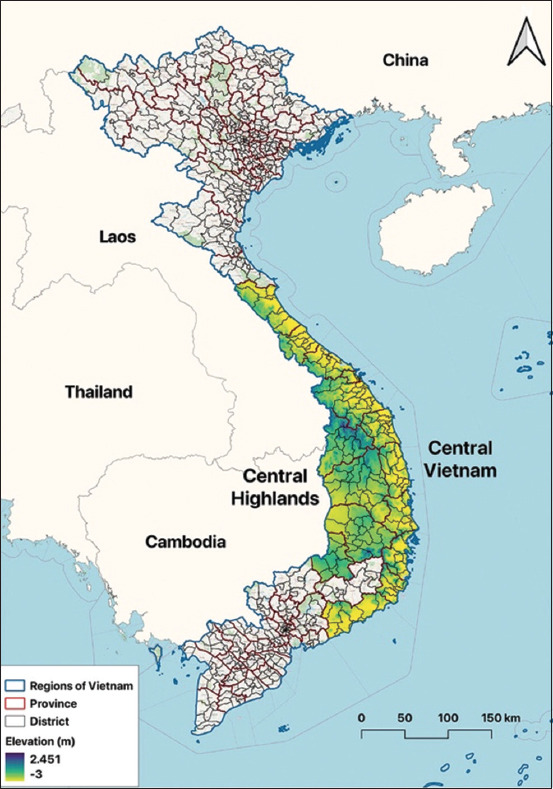
Map of the study site with administrative boundaries and adjacent nations. Color shading refers to elevation in the study area [Map was generated using Quantum GIS (QGIS) version 3.30 ‘s-Hertogenbosch (https://www.qgis.org/)].

The study was conducted from January 2014 to December 2018. The study was carried out in 15 Central Vietnamese provinces, encompassing 161 districts and 2,267 communes (Figure-1). In 2019, the region was inhabited by 17.9 million people [20]. Vietnam’s internal administrative boundaries were sourced from the Database of Global Administrative Areas (GADM), version 4.1, accessible at https://www.gadm.org.

Central Vietnam is situated between the North-west region and the Red River Delta to the north and the South-east region to the south. It lies between Laos and Cambodia to the west and the East Sea to the east. North Central Coast, Central Highlands, and South Central Coast are the three distinct topographical regions of the Central Region.

### Data collection

#### Data on Anopheles mosquitoes

**Figure-2:**
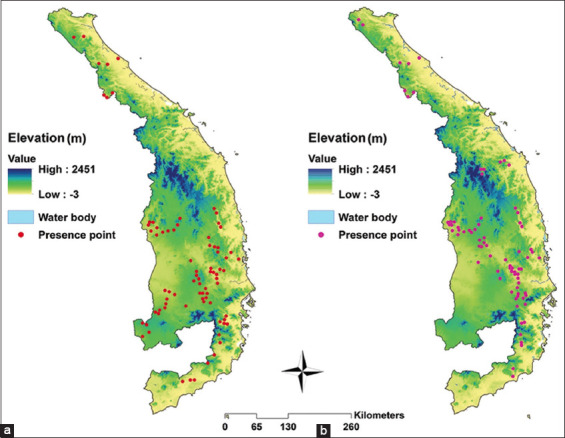
Presence points of (a) *Anopheles dirus* and (b) *Anopheles minimus* in Central Vietnam during 2014–2018 [Map was generated using Quantum GIS (QGIS) version 3.30 ‘s-Hertogenbosch (https://www.qgis.org/)].

From the Institute of Malariology, Parasitology, and Entomology (IMPE) Quy Nhon, Viet Nam database, records of *An. dirus* and *An. minimus* occurrences were obtained for the years 2014–2018. *Anopheles* spp. occurrence data with latitude and longitude were employed for modeling. The records were converted into geographic coordinates using the WGS84 Global Coordinate System (geocoding). This step was executed with Quantum GIS (QGIS) version 3.30 ‘s-Hertogenbosch [21]. Eighty occurrence points were recorded for *An. dirus*, while 78 for *An. minimus* (Figure-2).

#### Environmental, climatic, and socioeconomic data

The study employed diverse environmental, climatic, and socioeconomic proxy variables from 2014 to 2018. Land surface temperature (LST) data were acquired from Moderate Resolution Imaging Spectroradiometer (MODIS) MYD11A1 V6.1 sourced from the Aqua satellite, delivering high-resolution daily measurements (1 km) for both daytime and nighttime periods. Air temperature readings, specifically maximum air temperature (TMAX) and minimum air temperature (TMIN) at 2 m above the Earth’s surface, were obtained from European Centre for Medium-Range Weather Forecasts Reanalysis Fifth Generation Land (ERA5-Land). Normalized difference vegetation index (NDVI) calculations were based on Landsat 8 Collection 2 Tier 1 data, enabling monthly assessments of vegetation changes, with interpolation techniques applied to address missing data during high cloud cover instances. The study also incorporated the normalized difference water index (NDWI) derived from the MODIS Terra Daily NDWI satellite imagery, offering insights into vegetation moisture alterations. Monthly PREC values were derived from comprehensive Climate Hazards Group InfraRed Precipitation with Station data. Nighttime light (NTL) assessments were based on monthly composite radiance images downloaded from the visible infrared imaging radiometer suite day/night band, which was graciously provided by the Earth Observation Group at the Payne Institute for Public Policy, Colorado School of Mines.

A Google Earth Engine (GEE) script was designed to retrieve environmental, climatic, and socioeconomic proxy data for a specified region of interest that covered Central Vietnam. GEE encompasses a web-based interactive development environment alongside a JavaScript application programming interface. This combination grants users access to an extensive array of satellite products and data [22]. The data layers incorporated key factors that are hypothesized to influence the distribution range of *An. dirus* and *An. minimus* in Central Vietnam.

### Data processing

The raster data for the environmental, climatic, and socioeconomic variables mentioned above underwent projection, georeferencing, and resampling to achieve a consistent spatial resolution of 1 km × 1 km on the Earth’s surface. These data were converted into American Standard Code for Information Interchange (ASCII) format for analysis using MaxEnt. To extract the values of these environmental and climatic covariates for each *Anopheles* occurrence point, we used the point sampling tool in QGIS 3.30. In addition, we used the geographical extent of Central Vietnam in all maps.

### Variable selection and statistical analysis

We conducted a multicollinearity assessment to account for the inherent correlations among environmental, climatic, and socioeconomic variables. Any variables displaying a variance inflation factor exceeding 5 were excluded from subsequent analyses. Following this, we statistically evaluated the correlation between *Anopheles* spp. and each of the remaining environmental, climatic, and socioeconomic variables using a univariate logistic regression model. This analysis was performed using STATA 18.0 (Stata Corporation, College Station, Texas, USA). Any environmental, climatic, or socioeconomic variables with a Wald’s p > 0.2 were excluded from further consideration.

Next, we performed a backward stepwise multivariate logistic regression analysis with the remaining variables. As the criterion for inclusion, variables needed to have a significance level of p ≤ 0.05, and they were excluded if they had a Wald’s p > 0.1 [18, 23, 24].

### Species distribution models

MaxEnt software version 3.4.4 (accessible at http://biodiversityinformatics.amnh.org/open_source/maxent) was employed to develop potential distribution models for the species. For the MaxEnt analysis, 75% of the records were allocated as training data to build the model, while the remaining 25% were reserved for testing the model. The MaxEnt modeling process evaluates the significance of variables contributing to *Anopheles* spp. distributions through jackknife analysis, which assesses the contribution of each environmental, climatic, or socioeconomic variable to the model. Additionally, we computed the average values of the area under the curve (AUC) across 10 model iterations and determined the average percentage contribution of each variable to the model [25].

In terms of model accuracy, we used AUC values derived from receiver operator characteristic plot analysis within MaxEnt. An AUC value < 0.5 signifies a low predictive capacity for the model, whereas an AUC value nearing 1 indicates a high predictive potential [26].

## Results

**Figure-3:**
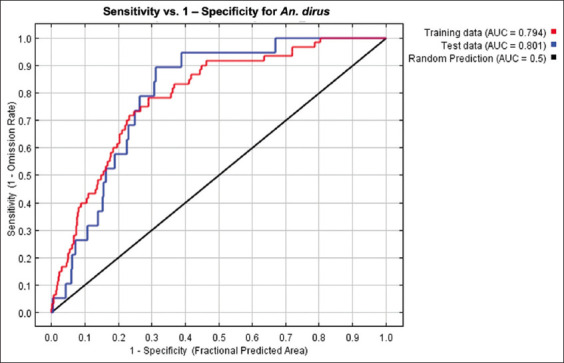
Receiver operating characteristic curve for MaxEnt runs for *Anopheles dirus*.

**Figure-4:**
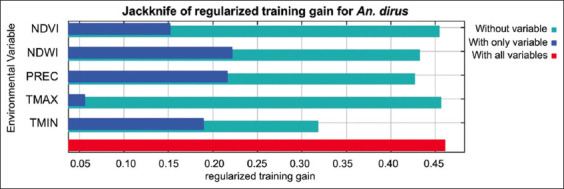
Relative predictive power of different variables based on jackknife evaluation of regularized training gain in MaxEnt models for *Anopheles dirus*.

**Figure-5:**
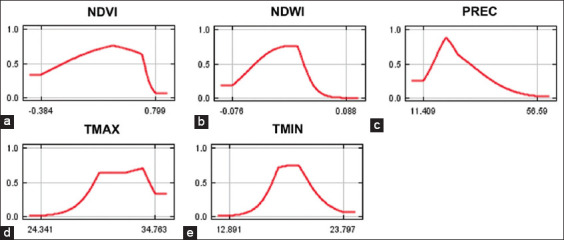
(a-e) Response curves showing the relationships between the probability of the presence of *Anopheles dirus* and climatic and environmental predictors. The value shown on the Y-axis is the predicted probability of the presence of *An. dirus*, while the X-axis shows the range of each predictor’s values.

The MaxEnt model for *An. dirus* reached an excellent score with an average test AUC value of 0.801 (Figure-3). The predicted distribution of *An. dirus* was significantly affected by TMIN (39.8%), PREC (20.4%), NDVI (19.3%), NDWI (18.5%), and TMAX (2%) (Supplementary Table-1). The jackknife evaluation of the *An. dirus* model showed NDWI to be the highest effective predictor when used individually, followed by PREC, TMIN, and NDVI. TMAX was the lowest effective predictor. The environmental variable that decreases the gain the most when it is omitted is TMIN (Figure-4). Analysis of the response curves shows that the presence of *An. dirus* has a negative correlation with PREC and NDWI (Figure-5).

**Supplementary Table-1:** Analysis of variable contributions in the MaxEnt model for *Anopheles dirus*.

Variable	Percent contribution	Permutation importance (%)
TMIN	39.8	53.3
PREC	20.4	16.1
NDVI	19.3	4.3
NDWI	18.5	22.5
TMAX	2	3.9

TMIN=Minimum air temperature, TMAX=Maximum air temperature, PREC=Precipitation, NDVI=Normalized difference vegetation index, NDWI=Normalized difference water index

**Figure-6:**
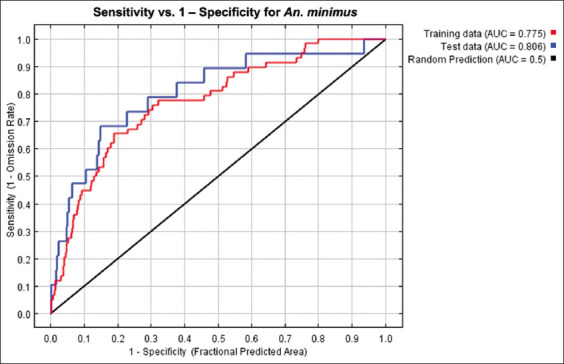
Receiver operating characteristic curve for MaxEnt runs for *Anopheles minimus*.

**Figure-7:**
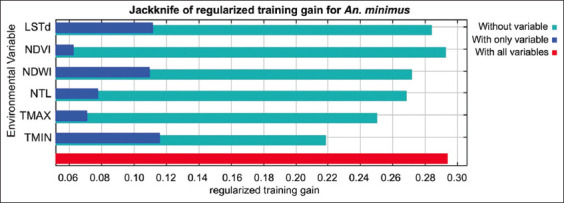
Relative predictive power of different variables based on jackknife evaluation of regularized training gain in MaxEnt models for *Anopheles minimus*.

**Figure-8:**
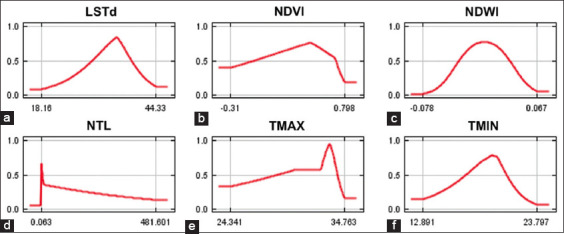
(a-f) Response curves showing the relationships between the probability of the presence of *Anopheles minimus* and climatic, environmental, and socioeconomic predictors. The value shown on the Y-axis is the predicted probability of the presence of *An. minimus*, while the X-axis shows the range of each predictor’s values.

The MaxEnt model for *An. minimus* yielded excellent results, achieving an average test AUC value of 0.806 (Figure-6). The predicted distribution of *An. minimus* was strongly affected by TMIN (36.3%), NDWI (21.1%), NTL (20.9%), TMAX (13.5%), LST daytime (LSTd) (4.5%), and NDVI (3.7%) (Supplementary Table-2). According to the jackknife evaluation of the *An. minimus* model, TMIN, LSTd, and NDWI were the three most effective predictors. The lowest effective predictor was NDVI. The climatic variable that decreases the gain the most when it is omitted is TMIN (Figure-7). The probability of *An. minimus* being present increased gradually as TMIN increased, starting at 13°C and peaking at 19.5°C before progressively declining, according to an analysis of the response curves. A negative link between the likelihood of *An. minimus* occurring and NTL (Figure-8).

**Supplementary Table-2:** Analysis of variable contributions in the MaxEnt model for *Anopheles minimus*.

Variable	Percent contribution	Permutation importance (%)
TMIN	36.3	49.7
NDWI	21.1	16.1
NTL	20.9	8.6
TMAX	13.5	21.6
LSTd	4.5	2.8
NDVI	3.7	1.1

TMIN=Minimum air temperature, TMAX=Maximum air temperature, NDVI=Normalized difference vegetation index, NDWI=Normalized difference water index, NTL=Nighttime light, LSTd=Land surface temperature daytime

**Figure-9:**
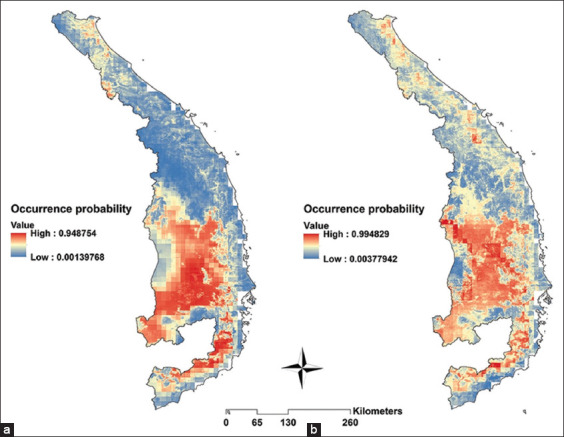
Potential distribution of (a) *Anopheles dirus* and (b) *Anopheles minimus* in Central Vietnam [Source: The Map was generated using Quantum GIS (QGIS) version 3.30 ‘s-Hertogenbosch (https://www.qgis.org/)].

Areas with high environmental suitability of *An. dirus* or *An. minimus* occurrence are shown in red, gradually decreasing to low probability in navy (Figure-9). Areas suitable for the distribution of both *An. dirus* and *An. minimus* are concentrated mainly in the Central Highlands, with some also in the North-Central and South-Central Coastal regions.

## Discussion

With MaxEnt modeling, we predicted the distribution patterns for two key malaria vectors in Central Vietnam. Our model relied on the IMPE Quy Nhon, Vietnam’s data (2014–2018) and utilized latitude and longitude coordinates as the primary input variables. The average test AUC values for *An. dirus* and *An. minimus* were 0.801 and 0.806, respectively, demonstrating the robustness of the derived MaxEnt models. These values indicate the models’ effectiveness in distinguishing appropriate from inappropriate habitats for the respective mosquito species. Distinct environmental predictors significantly influenced the distribution of these two species. *An. dirus* was influenced by TMIN, PREC, NDVI, NDWI, and TMAX while *An. minimus* was influenced by TMIN, NDWI, NTL, TMAX, LSTd, and NDVI.

Jackknife evaluations highlighted the importance of TMIN as a key predictor of both species, emphasizing the critical role of temperature in their habitat. Temperature-related factors have the highest influence on the distribution of *Anopheles* spp. [27]. A previous study by Obsomer *et al*. [28] showed that the maximum temperature is never too high (but remains above 24°C during the coldest month), while the minimum temperature is between 11°C and 22.5°C during the coldest month and between 19°C and 25°C during the warmest month provides ideal conditions for *Anopheles* spp.

Response curves demonstrated the correlation between environmental factors and the likelihood of mosquito occurrence. *An. dirus* and *An. minimus* showed negative associations with PREC and NTL. High monthly PREC levels make it possible to interpret the absence of mosquito larvae and pupae [29]. An adverse impact of increased rainfall in preceding months has been documented in Botswana [30] and Swaziland [31].

The distribution of *An. dirus* is significantly influenced by NDVI through multiple key mechanisms [8, 32]. High NDVI values typically signify dense vegetation, creating suitable habitats for this mosquito species because *An. dirus* favors sunlit pools amid dense vegetation [8], whereas *An. minimus* thrives in vegetated edges of water bodies [33, 34]. Conversely, lower NDVI values often correspond to areas with sparse vegetation, which might not support mosquito breeding because of inadequate resources or protection for larvae against predators, sunlight, and extreme temperatures [32].

Nighttime light is a reflection of urbanization and anthropogenic activity in general [35]. Extended human activity at night increases the chances for nocturnal mosquitoes to feed on humans, bypassing preventative measures like bed-nets [36–38]. In particular, NTL can attract mosquitoes, increasing the risk of mosquito bites and subsequent malaria transmission in areas with abundant artificial lighting at night [37]. Mosquitoes’ behavior is affected by nighttime lighting. It could interfere with their normal feeding and resting rhythms. Mosquitoes become more active at night, increasing their chances of infecting hosts and efficiently spreading diseases. Human behavior can be affected by nighttime light. Increases in human activity into the nighttime mean that nocturnal mosquitoes have a greater chance of obtaining a blood meal if they extend human activity outside of other prevention methods, such as bed-net use [37]. Our study found an inverse relationship between NTL and the occurrence of *An. minimus*. This trend is similar to the results of some other studies [36, 38] on *Anopheles gambiae* from Africa. Specifically, Das and Dimopoulos in 2008 [38] investigated the impact of white light on biting activity. Using a membrane feeding system and *An. gambiae* mosquitoes from an outbred strain found that even a brief exposure of 2 min to white light at intensities between 800 and 1000 lux during the late-night phase of the diel cycle significantly inhibited blood-feeding activity. This inhibitory effect persisted for up to 2 h after light exposure. Furthermore, Sheppard *et al*. [36] found that short exposures to light during the night or late in the day effectively inhibited biting activity and influenced the flight behavior of *An. gambiae*. To reduce the biting propensity throughout the night, the researchers developed a method involving repeated 6-or 10-min light pulses every 2 h. This approach suggests that light exposure could be a valuable supplementary tool alongside established malaria transmission control methods.

Analysis of spatial distribution showed that the Central Highlands of Vietnam had the highest concentration of areas with a high likelihood of occurrence for both *An. dirus* and *An. minimus*, while the North Central and South-Central Coastal areas had lesser, but still significant, concentrations. The research results facilitate personalized interventions based on identified behaviors and preferences. By concentrating our resources on these areas, we maximize the impact of vector control measures. These insights enable more accurate geospatial targeting, increased surveillance, and community outreach, improving our capacity to identify and address shifts in mosquito populations and disease transmission patterns. Prioritizing research in high-probability areas for mosquito species deepens our understanding and informs long-term control strategy development. The findings could reduce the impact of mosquito-borne diseases in affected communities and promote sustainable vector management methods.

MaxEnt modeling was used in this research to reveal substantial knowledge about *An. dirus* and *An. minimus* distribution in Central Vietnam. Acknowledging several limitations is necessary. The study’s data, covering the years from 2014 to 2018, may not accurately reflect long-term mosquito trends or seasonal variations. Mosquito population dynamics were explored, but factors such as natural enemies and public health control measures were not considered. Considering data quality and potential biases in our occurrence records is essential. Our data’s quality is considered high, due to several reasons. Entomologists skilled in mosquito collection use traps such as double net traps, cattle-baited traps, and Centers for Disease Control and Prevention miniature light traps for field sampling [6, 39]. *Anopheles* spp. were identified using dependable taxonomic keys or molecular techniques in the laboratory [40]. The presence of sporozoites in *Anopheles* mosquitoes’ salivary glands was confirmed through microscopic examination [41]. Robust quality-control measures, including peer review and result validation, instill confidence in the location data’s accuracy and subsequent collected samples’ identification [42].

The modeling approach itself has certain limitations. MaxEnt models assume equilibrium between species distributions and their environments, which may not always hold true, particularly in the context of changing climate conditions. The selection of predictor variables is somewhat arbitrary and influenced by data availability, and other relevant factors like land use, human population density, or ecological interactions are not considered [11].

## Conclusion

MaxEnt models accurately predicted the distribution of *An. dirus* and *An. minimus* in Central Vietnam, highlighting the importance of environmental factors. The minimum temperature significantly influenced *An. dirus* and *An. minimus*, with PREC and NDWI negatively correlated to *An. dirus* and a specific temperature range for *An. minimus*. Targeted malaria control efforts are crucial in the shared high-risk areas of the Central Highlands, North-Central, and South-Central Coastal regions. The study yields worthwhile conclusions but is constrained by its temporal frame (2014–2018), potential biases in occurrence data, and the assumed equilibrium in MaxEnt models. To effectively address malaria challenges in Central Vietnam and its high-risk areas, particularly considering climate change, research advancements, enhanced data collection, and advanced modeling techniques are needed.

## Data Availability

The original contributions presented in this study are included in the article/Appendix Material. Further inquiries can be directed to the first author and the corresponding author.

## Authors’ Contributions

LTT, ATS, KT, and SS: Study design, data extraction, statistical analysis, and drafted the manuscript. ATS and KT: Participated in critical editing of the manuscript. All authors have read, reviewed, and approved the final manuscript.
